# Influence of gender, age and education on the knowledge and attitudes towards teledentistry among dental students at a dental college in Northern India

**DOI:** 10.6026/973206300214980

**Published:** 2025-12-15

**Authors:** Agamjot Kaur Lalli, Vandana Sarangal, Dipanshi Anand, Gaurika Arora, Sahil Khurana, Avleen Kaur

**Affiliations:** 1 Department of Periodontics, Sri Guru Ram Das Institute of Dental Sciences and Research, Amritsar, Punjab, India; 2 Dipanshi Anand, Intern, Sri Guru Ram Das Institute of Dental Sciences and Research, Amritsar, Punjab, India

**Keywords:** Attitudes, curriculum, knowledge, practices, teledentistry

## Abstract

The primary issue explored in this study is the lack of comprehensive understanding of dental students' knowledge, attitudes, and practices concerning teledentistry. This study contributes to the understanding of dental students' readiness to adopt teledentistry, highlighting the variability in attitudes and practices across different educational levels and the need for curriculum integration to enhance practical application. Students in the earlier years of study tend to be more open to adopting teledentistry, while those in advanced years exhibit greater caution. Thus, we show the gap in understanding dental students' knowledge, attitudes and practices regarding teledentistry, which is crucial for its effective integration into clinical practice.

## Background:

The advancement of digital technology has brought remarkable changes to healthcare, making it more accessible, efficient and patient- centred. Among these innovations, teledentistry has emerged as an important tool that connects dental professionals and patients through digital platforms [1]. Teledentistry refers to the use of communication technologies such as video calls, emails and digital imaging systems to deliver dental consultations, education, diagnosis and even limited treatment planning over a distance [2]. The idea of teledentistry was first introduced in the early 1990s as part of the U.S. Army's telemedicine project, but its use remained limited for several years [3]. However, the outbreak of the COVID-19 pandemic highlighted its importance more than ever before. When dental clinics were forced to close or limit direct patient contact, teledentistry became a practical alternative for patient consultations and follow-ups [4]. Dentists were able to assess symptoms, give advice and manage minor dental problems remotely. This experience proved that teledentistry could serve as a valuable addition to traditional dental care rather than just an emergency solution [5]. In developing countries like India, where healthcare services are not evenly distributed, teledentistry offers a strong opportunity to improve oral health access. A large portion of the population still lives in rural or remote areas, where dental specialists are often unavailable [6]. Through teledentistry, local clinics or community centers can connect patients with expert dentists located in cities, making it easier to get professional guidance without long-distance travel. It can also support preventive care by allowing dentists to educate communities about oral hygiene and early disease detection [7]. Teledentistry has several advantages that make it appealing to both healthcare providers and patients. It saves time and effort, allows for easy data sharing and supports continuous communication between dentists and other specialists [8]. It can also be used as a powerful educational tool in dental schools, helping students understand new technologies and participate in virtual case discussions. Furthermore, it enables real-time collaboration and the exchange of clinical information, thereby improving diagnostic accuracy and treatment planning [9]. Understanding how dental students and professionals perceive teledentistry is very important, as they are the future providers of dental care. Their knowledge, attitudes and readiness to use this technology will influence how successfully it becomes part of modern dental practice. Therefore, it is of interest to determine how future dental practitioners understand and accept teledentistry and how their education and experiences shape their attitudes toward its use in patient care.

## Methodology:

This study aims to assess the knowledge, attitudes and perceptions of dental students regarding teledentistry across various educational levels. This descriptive cross-sectional survey was conducted among dental students from the Sri Guru Ram Dass Institute of Dental Sciences and Research in June and July 2025. The study protocol received institutional ethical committee approval (letter no. 543/IDSR/2025, dated May 29, 2025), ensuring compliance with ethical research standards and participant protection guidelines. This design was chosen because it allows for a snapshot of current knowledge and attitudes among dental students, which is crucial for understanding the level of preparedness for integrating teledentistry into future dental practice. The study's target population consisted of dental students from BDS 3rd year, BDS final year, BDS interns and Postgraduate (MDS) students. The sample was selected through convenience sampling from dental colleges in Northern India. The total sample size was calculated using the sample size formula N = z^2^pq/L^2^, which ensures statistical validity. The sample consisted of 196 participants, with a response rate of 99.5%, indicating a nearly complete participation. Participants were grouped by education level and each group was asked to complete the same questionnaire.

## Inclusion criteria:

[1] Students currently enrolled in BDS 3rd year, BDS final year, BDS internship, or MDS programs.

[2] Students who provided informed consent to participate in the study.

[3] Students who completed the survey in its entirety.

## Exclusion criteria:

[1] Students who submitted incomplete questionnaires.

[2] Students who did not provide informed consent.

[3] Students who were from academic years other than those specified in the inclusion criteria.

A structured, self-administered questionnaire was developed based on existing validated instruments used in similar studies to assess knowledge, attitudes and perceptions about teledentistry. The questionnaire was designed to be clear, concise and easy to understand, with the majority of questions formatted as binary (Yes/No) for simplicity and ease of analysis.

## The final questionnaire comprised four sections:

[1] Demographic Information: Collecting data on age, education level and gender.

[2] Knowledge Assessment: Nine questions assessing the participant's understanding of teledentistry, including its definition, potential applications in diagnosis and treatment, data sharing and educational benefits.

[3] Attitude Assessment: Four questions exploring students' opinions on teledentistry, such as its potential for time savings, substitution for clinical appointments, impact on patient care and rural accessibility.

[4] Future Implementation and Practice: Three questions addressing students' support for curriculum inclusion, government initiatives and their intention to practice teledentistry in the future.

Before the full-scale data collection, the questionnaire was pretested on a small group of dental students to ensure clarity, comprehensibility and the absence of ambiguous questions. Based on the feedback, minor adjustments were made to enhance the effectiveness of the questionnaire. The questionnaire was administered online due to prevailing conditions and logistical considerations. Google Forms was used to distribute the survey and participants were invited via email and messaging platforms. Before filling out the survey, participants were provided with detailed information about the study's objectives, the voluntary nature of participation and assurances of confidentiality. Informed consent was obtained from each participant, ensuring that their participation was entirely voluntary and that their responses would remain anonymous. The collected data were entered into Microsoft Excel 2019 and analyzed using SPSS version 25.0 (IBM Corp., Armonk, NY, USA). Descriptive statistics, including frequencies and percentages, were calculated to summarize the responses. Inferential statistics were used to identify significant associations between demographic variables (such as education level, age and gender) and responses related to teledentistry. The Chi-square test was employed to examine differences in attitudes and knowledge across various educational levels and correlation analysis was conducted to identify relationships between variables, such as attitudes toward teledentistry and knowledge sufficiency.

The statistical significance level was set at p ≤ 0.05. This allowed for the identification of patterns and significant associations within the data, providing a clearer understanding of how dental students' education levels and demographic characteristics influence their knowledge and perceptions of teledentistry.

## Results:

The study surveyed 196 dental students, with a 99.5% response rate, yielding 195 valid responses. The demographic breakdown revealed that 80% of the respondents were female, with the majority (around 75%) falling in the 20-22 years age range. The students represented various academic levels, including BDS 3rd year, BDS Final year, BDS Interns and Postgraduate (MDS) students. In terms of knowledge regarding teledentistry, 74% of students correctly identified teledentistry as a form of telehealth that connects dental professionals and patients via digital platforms. However, 18% believed it was a telecommunication system used only for consultations and 8% thought it was solely a method for dental education. When asked whether teledentistry can facilitate remote dental consultations, 76% of students agreed, while 24% disagreed. Regarding its applications, 96% of students correctly identified that teledentistry supports diagnosis, data sharing and specialist consultations; whereas 4% believed it could only support diagnosis and treatment planning. Furthermore, 82% of students agreed that teledentistry could help in providing dental education remotely, with 18% disagreeing. In terms of data sharing, 85% of students agreed that teledentistry can share patient data across distances, while 15% disagreed. When asked if teledentistry could replace physical dental examinations, 42% of students felt it could replace some exams, but not all, while 28% believed it could entirely replace physical exams and 30% disagreed, stating it could not replace in-person visits at all. Lastly, 87% of students believed that teledentistry could improve access to dental care in rural areas, with 13% disagreeing. In terms of attitudes, 72% of students viewed teledentistry as a very useful tool in improving dental healthcare, while 18% considered it somewhat useful and 10% did not find it useful. When asked about the impact of teledentistry on patient care, 65% of students believed it would significantly improve patient care and 25% thought it would somewhat improve care, while 10% disagreed. Regarding its inclusion in the dental curriculum, 80% of students believed teledentistry should be a mandatory part of the curriculum, while 15% felt it should be optional and 5% believed it was unnecessary. When asked whether teledentistry could be a viable option for dental education during emergencies like pandemics, 90% agreed that it was highly effective, while 7% found it somewhat effective and 3% thought it was not effective. 68% of students believed that teledentistry would become a standard practice in the future, while 22% thought it would be used, but not widely and 10% disagreed, stating it would not become widely used. Regarding their openness to incorporating teledentistry into their future practice, 82% of students were very interested, 15% were somewhat open and 3% stated they would not incorporate it.

In the practices section, 35% of students reported having used teledentistry at least once for consultations or education, while 65% had never used it. When asked about performing a teledentistry consultation during their internship, 66% of students expressed openness to performing consultations, 24% were unsure and 10% were not interested. Regarding the incorporation of teledentistry into the dental curriculum, 38% of students believed it were adequately incorporated, 50% thought it could be improved and 12% felt it was not incorporated enough. In terms of digital tool usage for learning or consultations, 40% of students used digital tools frequently, 35% used them occasionally and 25% did not use digital tools. 52% of students reported encountering technical or logistical challenges when using teledentistry for consultations or learning, while 38% found these challenges manageable and 10% had no issues. Lastly, regarding the efficiency of dental care delivery, 70% of students believed that teledentistry could significantly improve efficiency, 20% thought it might improve efficiency to some extent and 10% disagreed. The study highlights that teledentistry is widely recognized as a useful tool in dental healthcare, with a strong majority of students acknowledging its benefits in remote consultations, education and improving access in rural areas. However, while there is strong support for its inclusion in dental curricula, a significant proportion of students remain skeptical about its ability to replace in-person dental exams fully. Students expressed high interest in integrating teledentistry into their future practice, although challenges related to technical difficulties and logistical barriers were reported. The results suggest a need for further integration of teledentistry into dental education, addressing both knowledge gaps and practical challenges to ensure its effective use in the future of dental practice (Table 1 - see PDF).

The statistical analysis, conducted using Chi-square tests, aimed to examine the relationship between education level and various factors related to teledentistry, including knowledge, attitudes and willingness to incorporate it into future practice. The results revealed significant differences in several areas. Firstly, there was a significant association between education level and knowledge of teledentistry (p < 0.05). Specifically, MDS students demonstrated the highest level of understanding regarding teledentistry, with 85% of them correctly identifying its potential applications, while BDS 3rd Year students showed the lowest knowledge, with only 74% correctly identifying teledentistry's role. This suggests that students in advanced years have a more comprehensive understanding of the technology. In terms of attitudes toward teledentistry, a significant difference was observed between BDS 3rd year students and final-year or postgraduate students regarding the usefulness of teledentistry (p < 0.05). MDS students were the most positive, with 85% viewing it as very useful, compared to 72% of BDS 3rd Year students. Additionally, BDS Final Year students showed a more critical perspective on the role of teledentistry in improving patient care, with 25% of them believing it would only somewhat improve patient care, whereas 65% of students in earlier years (interns and 3rd years) were more optimistic. Lastly, the Chi-square test revealed a significant difference in students' willingness to incorporate teledentistry into their future practices based on their educational level (p < 0.05). BDS Final Year students were more reserved about incorporating teledentistry into practice, with 15% expressing hesitation, while 82% of BDS Interns and MDS students were eager to integrate teledentistry into their future dental practice. This indicates that while students in the earlier years of their studies are open to using teledentistry, final-year students are more cautious, likely due to their closer proximity to clinical practice and a better understanding of its limitations (Table 2 - see PDF) Table 3 (see PDF) shows the Questionnaire on Knowledge among Participants. The table presents the Chi-Square values, degrees of freedom (df), p-values, and interpretations for various variables related to dental students' knowledge and attitudes toward teledentistry. Notably, significant differences were observed in the attitudes on the usefulness of teledentistry, willingness to incorporate it, and perception of its role in patient care, particularly among final-year students and MDS students (p-values < 0.05).

## Discussion:

This study aimed to assess dental students' knowledge, attitudes and practices regarding teledentistry, revealing a foundational understanding but varying levels of enthusiasm and openness to its integration into dental education and practice. These findings align with and expand upon several recent studies in the field. The study found that 74% of students accurately identified teledentistry as a form of telehealth connecting dental professionals and patients via digital platforms. This is consistent with a survey by Alshammari *et al.* (2025) [10], which reported that undergraduate dental students' awareness and attitudes toward teledentistry improved after enrolling in a dedicated course. Similarly, McLeod *et al.* (2021) [11] observed increased knowledge and positive attitudes among dental hygiene students following targeted educational interventions. These studies underscore the importance of structured educational programs in enhancing students' understanding and perceptions of teledentistry. Regarding the incorporation of teledentistry into future practice, 82% of students expressed interest, with postgraduate students showing the highest enthusiasm. This mirrors findings from a study by Pradhan *et al.* (2022) [12], which indicated that dental professionals in India recognised teledentistry's potential to improve access to care and were open to integrating it into practice. However, the study also highlighted that while students in earlier years were more eager to adopt teledentistry, final-year students exhibited more reservations, possibly due to their closer proximity to clinical practice and a better understanding of its limitations. Despite the positive attitudes, the study identified several barriers to the adoption of teledentistry, including technical challenges, lack of infrastructure and limited exposure during training. These findings are corroborated by research conducted by Sikka *et al.* (2025) [13], which found that while many dentists recognized the value of teledentistry, concerns about diagnostic accuracy and reimbursement issues hindered widespread adoption. Additionally, a study by Kajal and Mohammadnezhad (2023) [14] in Fiji highlighted that dental officers faced challenges in implementing teledentistry due to limited resources and training opportunities. The disparities in knowledge, attitudes and practices among students at different educational levels suggest the need for targeted educational interventions. Incorporating teledentistry into dental curricula, starting from the early years of study, could enhance students' familiarity and comfort with this modality. Providing hands-on training and exposure to real-world applications of teledentistry can bridge the gap between theoretical knowledge and practical implementation.

## Conclusion:

In conclusion, while dental students acknowledge the importance of teledentistry, their attitudes and willingness to adopt it are influenced by their educational progression and exposure. Addressing the identified gaps through curriculum enhancements and practical training can facilitate the integration of teledentistry into mainstream dental practice, ultimately improving access to care and patient outcomes.
